# Development and Characterization of an HCMV Multi-Antigen Therapeutic Vaccine for Glioblastoma Using the UNITE Platform

**DOI:** 10.3389/fonc.2022.850546

**Published:** 2022-05-16

**Authors:** Amit S. Adhikari, Juliete Macauley, Yoshimi Johnson, Mike Connolly, Timothy Coleman, Teri Heiland

**Affiliations:** Immunomic Therapeutics, Rockville, MD, United States

**Keywords:** cancer vaccines, glioblastoma, immunotherapy, Th1 response, human cytomegalovirus, tumor microenvironment

## Abstract

Glioblastoma multiforme (GBM) is an aggressive form of brain cancer with a median survival of 15 months that has remained unchanged despite advances in the standard of care. GBM cells express human cytomegalovirus (HCMV) proteins, providing a unique opportunity for targeted therapy. We utilized our UNITE (UNiversal Intracellular Targeted Expression) platform to develop a multi-antigen DNA vaccine (ITI-1001) that codes for the HCMV proteins pp65, gB, and IE-1. The UNITE platform involves lysosomal targeting technology, fusing lysosome-associated membrane protein 1 (LAMP1) with target ntigens. We demonstrate evidence of increased antigen presentation by both MHC-I and -II, delivering a robust antigen-specific CD4 and CD8 T-cell response in addition to a strong humoral response. Using a syngeneic orthotopic GBM mouse model, therapeutic treatment with the ITI-1001 vaccine resulted in ~56% survival of tumor-bearing mice. Investigation of the tumor microenvironment showed significant CD4 infiltration as well as enhanced Th1 and cytotoxic CD8 T activation. Regulatory T cells were also upregulated after ITI-1001 vaccination. In addition, tumor burden negatively correlated with activated interferon (IFN)γ+ CD4 T cells, reiterating the importance of CD4 activation in ITI-1001 efficacy and in identifying treatment responders and non-responders. Further characterization of these two groups showed high infiltration of CD3+, CD4+, and CD8+ T cells in responders compared to non-responders. Thus, we show that vaccination with HCMV antigens using the ITI-1001-UNITE platform generates strong cellular and humoral immune responses, triggering significant antitumor activity, leading to enhanced survival in a mouse model of GBM.

## Introduction

Glioblastoma multiforme (GBM) is an aggressive brain cancer with 5-year survival rates below 10% (1). Current treatment includes surgical removal of the tumor followed by chemotherapy and/or radiation therapy; however, successful treatment only increases the median survival to ~15 months (1, 2). This standard-of-care treatment has remained unchanged for decades, creating an urgent need for novel treatments for this disease. Human cytomegalovirus (HCMV) proteins were first shown in GBM tumors in 2002 and were later confirmed by several groups (3–6). Having said this, several studies and major consortia were unable to detect the presence of CMV transcript and/or protein in glioblastoma tissue. In an attempt to address this controversy, HCMV and glioma symposium was convened in 2011, and a consensus was reached that there is sufficient evidence to conclude that HCMV sequences and gene expression exist in most, if not all, malignant gliomas (7). The precise role of HCMV in the GBM pathogenesis remains unclear. From a therapeutic perspective, the specific presence of HCMV antigens in more than 90% of GBM tumors provides an opportunity to develop a targeted therapy for GBM while simultaneously harnessing preexisting antiviral immunity (4).

It has been previously shown using our lysosome-associated membrane protein (LAMP) fusion platform that autologous dendritic cells (DCs) pulsed with LAMP-pp65 mRNA significantly improve survival in GBM patients, providing clinical validation for this strategy (8, 9). Though autologous DC immunotherapy using LAMP-pp65 mRNA demonstrates the effectiveness of HCMV-targeted therapy and has great potential in the clinic, there are some limitations of this treatment. It is highly personalized and requires professional personnel to collect and transfect the DCs under aseptic conditions, thereby increasing the cost of treatment and limiting the availability to specialized hospitals. In addition, certain patients due to their medical condition are not suitable for leukapheresis and hence ineligible for autologous DC immunotherapy.

To overcome these limitations of the autologous DC therapy, we utilized our UNITE (UNiversal Intracellular Targeted Expression) platform to develop a multi-antigen DNA vaccine (ITI-1001) encoding HCMV antigens. The major component of the UNITE platform involves fusion of HCMV antigens with LAMP1. LAMP1 is a glycosylated protein that is transported through the trans-Golgi network into lysosomes and then attached to the internal lysosome membrane through the C-terminus (10, 11). It has been previously shown that the fusion of LAMP1 with antigens induces preferential CD4-mediated T-cell immune responses through the MHC-II–lysosome pathway (12–14). Additionally, the UNITE platform drives CD8 T-cell activation *via* the MHC-I pathway. Specifically, antigen-LAMP1 fusion construct displayed a significantly higher percentage of CD8 T cells secreting interferon (IFN)γ and tumor necrosis factor (TNF)α leading to better survival compared to construct with antigen alone in a breast cancer mouse model, indicating the advantage of using UNITE platform (14). The ITI-1001 vaccine contains three HCMV antigens: phosphoprotein 65 (pp65; UL83), immediate-early protein 1 (IE-1; UL123), and glycoprotein B (gB; UL55); pp65 and gB are structural proteins, whereas IE-1 is a transcription factor (15–17). The presence of all three antigens has been confirmed in the majority of GBM tumors by Western blotting and immunohistochemistry (4, 5). In addition, all three proteins are highly immunogenic, inducing strong T-cell responses and are considered promising candidates for HCMV vaccine development (18). pp65-specific CD8 and CD4 T cells have been identified in GBM patients, further confirming the immunogenicity of this antigen and preexisting antitumor T-cell repertoire in patients (19). Taking advantage of the presence of HCMV-specific T cells in GBM patients, researchers have isolated and enriched these autologous T cells and succesfully demostrated improved overall survial in primary GBM patients (20–22). Together, these observations provide a strong corroboration for using HCMV antigens to develop a multi-antigen vaccine for GBM.

In the present study, using ITI-1001 vaccination of naive mice, we demonstrated strong antigen-specific humoral response and T-cell activation in both CD8 and CD4 T-cell compartments as evaluated by their ability to secrete high levels of IFNγ and TNFα. Therapeutic vaccination with ITI-1001 conferred more than 50% survival in an orthotopic syngeneic GBM mouse model expressing HCMV antigens. Furthermore, based on our tumor microenvironment (TME) studies, ITI-1001-treated tumors showed significant CD4 T-cell infiltration and activation and enhanced activation of CD8 T cytotoxic killer cells, along with an increase in Natural Killer T (NKT) cells thereby providing possible clues into the mechanism(s) of action of ITI-1001. Interrogating the bimodal separation of activated CD4 T cells and their negative correlation with tumor burden identified responders and non-responders of the ITI-1001 treatment group. Further characterization of responders showed higher TME infiltration of both CD4 and CD8 T cells along with increased CD8 T-cell activation. Taken together, our data show that ITI-1001 generates strong cellular and humoral immune responses, triggering significant antitumor activity that leads to enhanced survival in this preclinical evaluation of the vaccine.

## Materials and Methods

### Vector Construction

ITI-1001 is composed of two plasmids: L-H-IE1-pp65co, containing a fusion of IE-1 and pp65 antigens with LAMP, and L-H-CMV-gB plasmid, where gB antigen is fused with LAMP1 in the NTC8382-VA1 vector. These were specifically designed as safe minimalized antibiotic-free selection vectors (Nature Technologies, Lincoln, NE). The vectors combine minimal prokaryotic sequences including an antibiotic-free sucrose selectable marker. These also contain a novel chimeric promoter that directs superior mammalian cell expression. Consistent with the Food and Drug Administration (FDA)‘s regulatory guidance regarding DNA vaccine vector composition, all sequences that were not essential for *Escherichia coli* plasmid replication or mammalian cell expression of the target gene were eliminated. Synthetic eukaryotic mRNA leader and terminators were utilized in the vectors to limit DNA sequence homology with the human genome to reduce the possibility of chromosomal integration (23). In addition, the pp65 sequence was codon optimized to enhance expression in humans.

### Naive Mouse Immunization and Blood Collection

Six- to eight-week-old female C57BL/6 mice were purchased from Jackson Laboratories and maintained at the animal facility at Immunomic Therapeutics, Inc. All animal studies were performed according to Institutional Animal Care and Use Committee (IACUC) guidelines. Mice were immunized weekly with 40 μg of ITI-1001 (20 μg of each plasmid) *via* intradermal (ID) injection in the mouse ear pinna followed by electroporation (EP) using the ICHOR TriGrid Delivery System, model-TDS II, weekly for 4 weeks. Serum was collected prior to immunization (prebleed) and 3 days after the last immunization by submandibular bleeds. For mice receiving the ITI-1001 product, the two plasmids (L-H-IE-1-pp65co and L-H-CMV-gB) were mixed at equal concentrations (the specific ITI-1001 concentration was dependent on the specific study). For the control group, control vector plasmid (NTC8382-VA1, vector backbone alone) was given at the same concentration as ITI-1001.

### Transfection, Degylcosylation, and Western Blot

In this study, 293T cells were transfected with 5 µg ITI-1001 using Lipofectamine 2000 (Thermo Fisher Scientific, 11668019). Cells were transfected with 5 µg of hLAMP1 pDNA or no pDNA as positive and negative control, respectively. Cell extracts were prepared using radioimmunoprecipitation assay (RIPA) buffer (Thermo Fisher Scientific, 89900) containing HALT protease inhibitor cocktail (Thermo Fisher Scientific, 78430). Here, 50 µg of cell extract was used for deglycosylation (NEB, B6045S), deglycosylation mix II (NEB, P6044S). Also, 5 µg of untreated and treated (deglycosylated) cell extracts were electrophoresed using 7.5% TGX gels (Bio-Rad 4561023) and blotted to 0.2 µM polyvinylidene difluoride (PVDF) membranes (Bio-Rad 1704156) using a Bio-Rad Turbo Blotter. The membranes were probed with specific antibodies (anti-IE1, Light Diagnostics; MAB810, anti-pp65, Invitrogen; MA17597 and anti-gB, Sino Biologicals, 10202-R038) and anti-Glyceraldehyde 3-phosphate dehydrogenase (GAPDH) antibody (Invitrogen, MA5-15738) using an iBind system (Invitrogen). The membranes were detected using Clarity Western ECL reagent (Bio-Rad, 10231-400/1020313999), and images were acquired using Syngene G-Box mini system with GeneSys software.

### ELISpot

Spleens were collected 10–14 days post vaccination, crushed, filtered using 0.44-μM filters, and red blood cells were lysed using TONBO RBC lysis solution (Ack lysis buffer, TNB-4300-L100). Here, 350,000 live splenocytes were seeded per well for 48 h containing pp65 (JPT, PM-PP65-1), IE-1 (JPT, PM-IE-1), and gB (JPT, PM-UL55) peptides (1 μg/ml of each peptide mix) or concanavalin A (ConA, 2.5 μg/ml, Sigma, C5275) as a positive control on ELISpot plates (Millipore, MSIPS4510). Cells with media alone were used as a negative control. Results shown after reducing the background value in media only wells for each mouse. Antibody pairs for IFNγ [BioLegend, coating antibody 517902 (clone AN18), biotinylated antibody 505704 (biotin R46A2)], for interleukin (IL)-4 [BioLegend, coating antibody 504108 (clone 11B11), biotinylated antibody 505202 (biotin BVD624G2)], for IL-10 [Mab Tech, coating antibody 3432-3 (clone MT60), Biotinylated antibody 3432-6 (biotin 51E6)]. The ELISpot plates were developed using BCIP/NBT-plus reagent from MabTech (3650-10) after incubating with streptavidin-ALP Ab (MabTech, 3310-8). To calculate total antigen-specific immune response for all three antigens together, individual antigen-specific spots were summed for each mouse.

### ELISA

For antibody (Ab) titer analysis, serum Abs were tested post vaccination. ELISA was done with serum concentration range 1:100 to 1:100,000 for gB, pp65, and IE-1 total Abs. ELISA plates were coated with 5 μg/ml concentration for each protein (total of 0.5 μg protein/well), pp65 protein, Nature Inc., gB (Sino Biologicals, 10202-V08H1), IE-1 protein (My Biosource, MBS505046 and Genway Biotech Inc., GWB-MA0541). Antigen-specific total IgG antibody was detected using Horseradish peroxidase (HRP)-conjugated anti-mouse antibodies (Southern Biotech, 10-30-05) and colorimetric signal obtained by reading the plates at 650 nm (BioTek, Epoch spectrophotometer).

### Intracellular Cytokine Staining and Analysis Using Flow Cytometry

Splenocytes were collected 10–14 days after vaccination and incubated with antigen-specific peptides for a total of 5 h (1 h with antigens and then brefeldin A and monesin were added for an additional 4 h), and T-cell panel antibodies (more information in **Supplementary Table S1**) were used for staining followed by flow cytometry using Cyto FLEX (Beckman Coulter). Fluorescence minus one (FMO) controls were used to define gates for various populations. Data were analyzed using Kaluza Analysis software 2.1 (Beckman Coulter). Unpaired Student’s t-test was used for statistical analysis using GraphPad Prism software.

### Human Cytomegalovirus-Glioblastoma Multiforme Syngeneic Orthotopic Mouse Model and Efficacy Study

Mouse GBM cell line CT2A (gifted by Dr. John H. Sampson) was transduced sequentially using lentivirus with pp65-IE-1-luciferase fusion followed by gB-luciferase fusion. Puromycin and hygromycin respectively were the selection markers for the two fusions used to make a stable cell line expressing the three HCMV proteins. Multiple clones were tested, and the number of cells injected were optimized to reduce the autologous immune response to the three exogenous HCMV antigens. Stable clones were cultured in 50% of the lethal antibiotic dose, and 40,000 cells were injected intracranially using stereotaxic apparatus (Quintessential Stereotaxic Injector, Stoelting, 53311). Coordinates—from bregma 2 mm right and 5 mm depth. The mice were under isoflurane anesthetic, local anesthetic bupivacaine, and meloxicam was used as an analgesic, during the intracranial injection. Bone wax was used to seal the hole made in the skull and wound clips to seal the cut skin. Tumors were monitored twice weekly using luciferase *in vivo* bioluminescence imaging (IVIS LuminaLT Series III, Perkin Elmer) using XenoLight D-Luciferin Potassium Salt (Perkin Elmer, 122799) injected intraperitoneal (IP) as a substrate. Mice were euthanized after they showed debilitating neurological symptoms or excessive weight loss, and necropsy was conducted. For efficacy studies, the mice were vaccinated four times weekly.

### Tumor Microenvironment Study

Mice were intracranially injected with 70,000 CT2A cells expressing the three HCMV antigens and monitored for tumor growth. When most of the mice showed a bioluminescence signal, two vaccinations of ITI-1001 were given weekly. Mice were euthanized 1 week post second vaccination, and tumors were collected, weighed, and dissociated into single-cell suspension using Miltenyi Biotec tumor dissociation kit (130-096-730), gentleMACS C tubes (Miltenyi Biotec 130-096-334), and Miltenyi Biotec dissociator. Cells were stained for 6 different panels, T cells, regulatory T cells (Treg) and Programmed cell death protein 1 (PD-1)-expression, DCs, NK cells, M1 and M2 macrophages, and Myeloid Derived Suppressor cells (MDSC) surface markers. For intracellular markers, cells were fixed using Foxp3/Transcription Factor Staining Set (eBioscience 00-5523-00). Antibody details are given in **Supplementary Table S1**. Zombie Aqua was used to detect live and dead cells and UltraComp eBeads (eBioscience 01-2222-42) on Cyto FLEX, Beckman Coulter, for compensation. FMO controls were used to define gates for various populations. Data were analyzed using Kaluza Analysis software 2.1 (Beckman Coulter). Unpaired Student’s t test was used for statistical analysis using GraphPad Prism software. **Table 1** shows responder, non-responder statistical analysis. We performed 3 studies to evaluate the response within the TME to ITI-1001 treatment.

**Table 1:** Average frequencies of T-cell populations and tumor weight in responders and non-responders with statistical significance.

	cells/g of tumor Average values with n = 6		P value * ≤=0.05 and ***≤=0.0005	
Cell population	Responders	Non-responders		
CD45+	27,495,701	30,743,102	0.596282	
CD3	4,011,947	1,811,957	0.011510	*
CD4	1,294,400	466,948	0.000474	***
CD8	2,162,425	999,934	0.064209	
CD8 Effector Memory (TEM)	2,001,300	918,311	0.077122	
CD8 Interferon γ	312,051	64,874	0.014638	*
CD8 Tumor necrosis factor α	50,207	32,980	0.342217	
CD8 Granzyme B	635,512	116,993	0.052007	
CD4 Interferon γ	167,338	21,040	0.000005	***
CD4 Tumor necrosis factor α	89,474	27,766	0.048874	*
Tumor weight	0.037	0.19	0.018534	*

Different cell populations were compared for cell numbers per gram of tumor between responders and non-responders. p values from Student’s t-test are denoted to signify the statistical differences between responders and non-responders.

p-*=<0.05, ***=<0.0005.

### Cytokine Evaluation Using Meso Scale Discovery Assay

As described in the TME study, mice with tumors were treated with ITI-1001 and control plasmid. After two vaccinations, mice were euthanized 4 days after the second vaccination, and tumors were collected and weighed. Tumors were lysed using lysis solution with protease inhibitor cocktail (Meso Scale Discovery, R60TX-2, R70AA-1) and Minilys homogenizer and Bertin-corp.com, P000922-LYSKO-A.0, CKMix5-R, 2 ml tubes. Protein concentrations were determined using Bicinchoninic Acid (BCA) Protein Assay, and 100 μg of tumor lysate was used in duplicate in the V-PLEX Proinflammatory Panel 1 Mouse Kit (Meso Scale Discovery, K15048D). Plate signals were read using MESO QuickPlex SQ 120, and data were analyzed using Meso Scale Discovery, Discover Work Bench software. Student’s t-test was used to determine the statistical significance using GraphPad Prism software.

## Results

### ITI-1001 Shows Robust Cellular and Humoral Immune Response in Naive Mice

**Figure 1:**
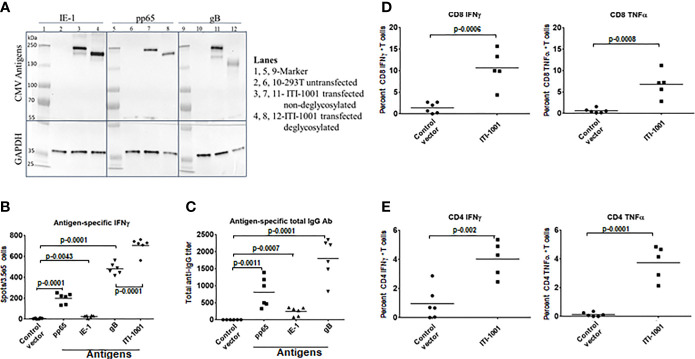
Expression and immunological response of ITI-1001 vaccine. **(A)** Western blots showing the expression of IE-1, pp65, and gB HCMV antigens from ITI-1001 in 293T cell lysates using antigen-specific antibodies. Lanes 2, 6, and 10 are untransfected cell lysates. Lanes 3, 7, and 11 are ITI-1001-transfected non-deglycosylated cell lysates. Lanes 4, 8, and 12 are ITI-1001-transfected deglycosylated cell lysates. Glyceraldehyde 3-phosphate dehydrogenase was used as a loading control. Molecular weight ladder was used in lanes 1, 5, and 9 to assist in protein size determination. **(B)** IFNγ ELISpot data with antigen-specific peptides for pp65, IE-1, and gB and all three antigens combined using splenocytes. p values from Student’s t-test are denoted to signify the statistical differences between the groups. **(C)** Serum-based Enzyme linked immunosorbent assay for antigen-specific total antibody detection. p values from Student’s t-test are denoted to signify the statistical differences between the groups. **(D, E)** Flow cytometric analysis of CD8 and CD4 T cells respectively for antigen-specific activation and intracellular cytokine staining for IFNγ and TNFα. The percent T cells in each graph are from T effector memory cell populations (CD44^+^ CD62^-^) for CD4 and CD8 T cells. p values from Student’s t-test are denoted to signify the statistical differences between the groups.

To evaluate the expression of the three HCMV antigens (pp65, IE-1, and gB) encoded by the two plasmids that comprise ITI-1001, human embryonic kidney 293 cells (293T cells) were transiently transfected with L-H-IE-1-pp65co and L-H-CMV-gB plasmids, and protein extracts were used for Western blot analysis. As shown in **Figure 1A**, IE-1, pp65, and gB proteins were detected both by antigen-specific antibodies and a LAMP-specific antibody (**Figure 1A**, lanes 3, 7, and 11, and **Supplementary Figure S1**, respectively). Due to the heavy glycosylation of LAMP1, we find higher-than-expected molecular weights of the fused antigens. Deglycosylation of the 293T cell lysates (**Figure 1A**, lanes 4, 8, and 12) lowered the molecular weights to the expected molecular weight of the antigens (IE-1-pp65-LAMP1 fusion ~213 kDa and gB-LAMP1 fusion ~202 kDa) there by confirming the expression of these antigens with LAMP1 fusion from the vaccine plasmids. GAPDH, a housekeeping protein, was used as an internal loading control. Lanes 2, 6, and 10 were loaded with lysates from untransfected 293T cells, a negative control, and as expected did not show any protein band.

**Figure 1B** shows antigen-specific IFNγ expression, by ELISpot, indicative of a pro-inflammatory antitumor Th1 response. gB showed the highest IFNγ spots followed by pp65 and then IE-1. The Th2 response cytokines IL-4 and IL-10 (anti-inflammatory/immunosuppressive markers) were also evaluated, and both cytokines showed an insignificant number of spots (**Supplementary Figure S2**, IL-10 data not shown), suggesting that the ITI-1001 vaccine does not elicit these pathways in naive mice. We also found a strong antigen-specific humoral response, as seen in **Figure 1C**, with similar trends as the ELISpot data where gB, pp65, and IE-1 showed the humoral response highest to lowest in the same order (gB > pp65 > IE-1). To understand the T-cell subtype involved in the IFNγ production observed in ELISpot, we performed *in vitro* activation of splenocytes from vaccinated naive mice using antigen-specific peptides followed by intracellular staining and flow cytometry. **Figures 1D, E** show that both CD8 and CD4 T cells were activated by antigen-specific peptides when probed for IFNγ and TNFα pro-inflammatory cytokines.

### ITI-1001 Shows More Than 50% Survival in an Orthotopic Syngeneic Glioblastoma Multiforme Mouse Model

**Figure 2:**
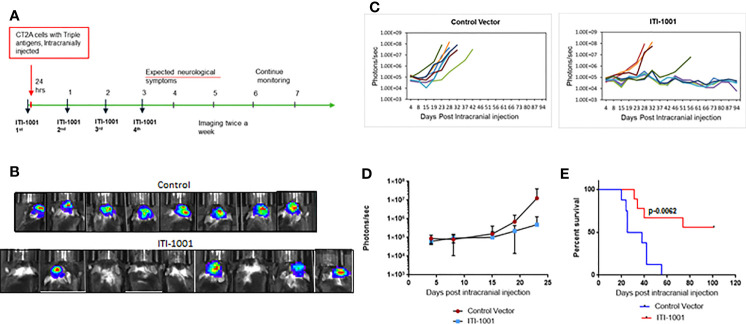
ITI-1001 confers survival benefit in an orthotopic syngeneic GBM mouse model. **(A)** Schedule for efficacy study with intracranial injection, vaccinations, and imaging information. **(B)** Representative bioluminescence images of mice injected with CT2A cells expressing the three human cytomegalo virus antigens in control and ITI-1001-treated group and the maximum signal shown by each mouse. **(C)** Tumor growth kinetics plotted using bioluminescence signal and the days post intracranial injection for control and ITI-1001 group. **(D)** Average bioluminescence is shown in the third plot for the time points where all the mice were alive. **(E)** Survival curve for control and ITI-1001-treated group plotted with percent survival and days post intracranial injection. Control group (n = 8) had a median survival of 31.5 days, and ITI-1001 (n = 9) median survival could not be calculated due to insufficient deaths. p value (0.0062) from log-rank (Mantel–Cox) test denotes the statistical difference between the two curves. The percent survival was 56%.

In order to evaluate the therapeutic efficacy of ITI-1001vaccine in a mouse GBM model, we developed an orthotopic syngeneic GBM mouse model using the mouse CT2A cell line stably transfected with the HCMV genes encoding pp65, IE-1, and gB. The CT2A GBM model in syngeneic mice has been demonstrated to closely mimic human GBM disease (24). This model has a low mutational burden and is known to be refractory to checkpoint inhibitor monotherapy like human GBM (25). The gene encoding the luciferase enzyme was fused with the HCMV antigens, and tumor growth was measured by bioluminescence imaging. **Figure 2A** shows the schedule of the therapeutic efficacy study. We chose the intradermal route of administration for delivery of our multiantigen vaccine, as the dermal space is known to be populated with abundance of antigen-presenting cells (APCs), mainly DCs and Langerhans cells. We expect these cells to get transfected and present HCMV antigens preferentially on MHC-II and also on MHC I. **Figure 2B** shows representative images of individual max bioluminescence signal in control and ITI-1001-treated groups. **Figure 2C** shows the tumor growth kinetics of individual mice in the control and the ITI-1001-treated groups. In the control group, all 8 mice showed tumor growth compared to only 4 mice in the ITI-1001 treatment group out of a total of 9 mice. The tumor growth kinetics and bioluminescence intensities of non-responders in the ITI-1001-treated group and control group were similar. Average bioluminescence observed when all mice were alive showed approximately one log difference, with the control group having a higher signal intensity compared to that in the treated group, shown in **Figure 2D**. Survival curves indicate that the control group had a median survival of 30 days, and all the mice were dead by day 41. In the group treated with ITI-1001 vaccine, we observed that 5 mice out of 9 mice survived (56%), and due to the fewer number of deaths, the median survival could not be determined, shown in **Figure 2E**.

### Effect of ITI-1001 Vaccination on the Immune Responses Within the Tumor Microenvironment to Understand the Mechanism of Action and Identify Responders and Non-Responders

**Figure 3:**
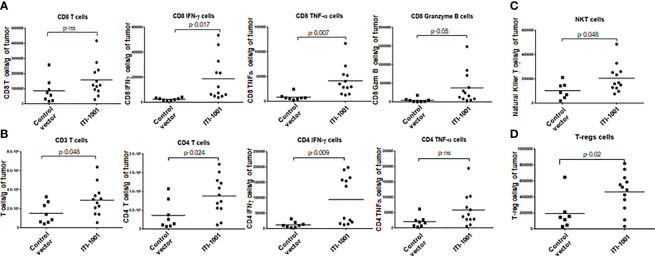
ITI-1001 activates CD4, CD8, and NK T cells in the tumor microenvironment (TME) along with immunosuppressive regulatory T cells. **(A)** Flow cytometric data for total and activated CD8 T cells in the TME with and without ITI-1001 vaccination. For CD8 T-cell activation, intracellular staining of IFNγ, TNFα, and granzyme B are shown. **(B)** Flow cytometry data for total T cells and total and activated CD4 T cells in the TME with and without ITI-1001 vaccination. For CD4 T-cell activation, intracellular staining of IFNγ and TNFα is shown. **(C, D)** Flow cytometry data showing natural killer T cells and Tregs respectively in the TME from the two groups. All the populations are denoted as cell numbers per gram of tumor. p values from Student’s t-test are denoted to signify the statistical differences between the control and treated tumors.

In order to gain insight into the mechanism of action of ITI-1001 vaccine, we investigated the TME with and without the treatment of the vaccine. **Figures 3A, B** show the data from the T-cell panel; Panel A shows CD8 T-cell infiltration and activation. We did not observe any difference between the control and treated group regarding the infiltrating CD8 T cells. In terms of activated CD8 T cells, we found that all three cytokines, IFNγ, TNFα, and granzyme B, were significantly expressed in the ITI-1001-treated group compared to those in the control group. Interestingly, Panel B shows an increase in the number of CD4 T cells, infiltrating in treated tumors compared to control, along with total CD3 T cells. In addition, CD4 T cells showed strong IFNγ expression, indicating their immunologically active status, whereas TNFα expression was comparable to that in the control group. In addition to T-cell activation, we also observed NKT cells in higher numbers in treated tumors (**Figure 3C**). Furthermore, we also investigated the potential immunosuppressive mechanism in the TME after ITI-1001 administration by probing into PD-1, Treg, MDSCs, and M1/M2 macrophage populations. **Figure 3D** and **Supplementary Figure S3** show that only Treg population was significantly upregulated in the tumors from vaccinated mice. **Supplementary Figure S5A** shows the gating strategy for all 5 panels, and **Supplementary Figure S5B** shows the FMO controls.

**Figure 4:**
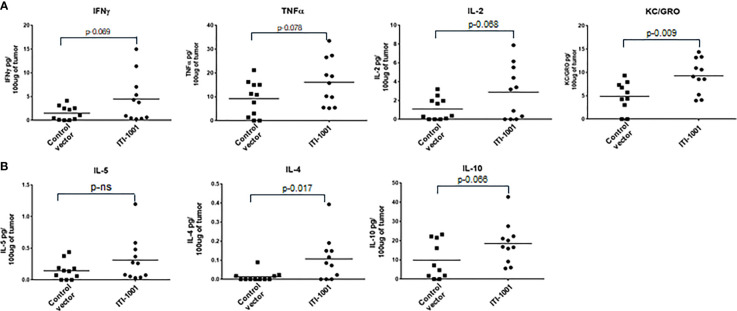
Evaluating TME cytokine levels. **(A)** Cytokine levels denoted as pg/100 μg of tumor lysate for Interferonγ, tumor necrosis factorα, IL-2, and Keratinocyte Chemoattractant/human Growth-Regulated Oncogene. **(B)** Cytokine levels for IL-5, IL-4, and IL-10 denoted as pg/100 μg of tumor lysate. p values from Student’s t-test are denoted to signify the statistical differences between the control and treated tumors.

When we evaluated the cytokine expression in the TME using Meso Scale Discovery (MSD) assay, we found that the pro-inflammatory cytokines such as IFNγ, TNFα, IL2, and Keratinocyte Chemoattractant/human Growth-Regulated Oncogene (KC/GRO) were upregulated in ITI-1001-treated tumors (p ≤ 0.1), supporting the tumor T-cell activation data observed by flow cytometry. In addition, anti-inflammatory (immunosuppressive) IL-10 and the Th2 cytokine IL-4 were also upregulated in the treated tumors, as shown in **Figure 4**. Another Th2 cytokine IL-5 did not show any difference between expression in both control and treated group.

**Figure 5:**
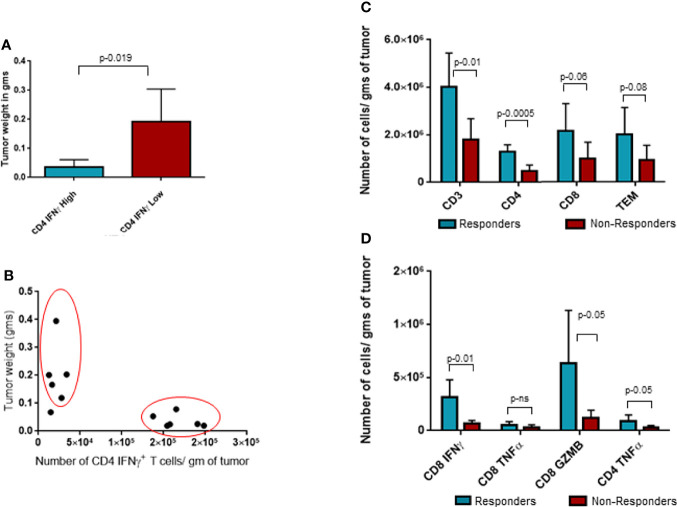
Analysis of responders and non-responders based on CD4 Interferonγ expression levels. **(A)** Tumor weight data in grams for two groups of ITI-1001-treated tumors based on the two populations of CD4 Interferonγ high and low levels observed in **Figure 3B**. **(B)** Unbiased correlation plot between tumor weight and CD4 Interferonγ+ samples. Correlation coefficient r-(-0.71). **(C)** Evaluating the differences between the responders and non-responders for T-cell population. **(D)** Evaluating the differences between the responders and non-responders for activated CD8 and CD4 T cells using IFNγ+ and TNFα+ expression. All the populations are denoted as cell numbers per gram of tumor. p values from Student’s t-test are denoted to signify the statistical differences between responders and non-responders.

Upon closer observation, it was seen that CD4+IFNγ+ population was clearly segregated in two populations based on the number of these cells per gram of tumor. We took advantage of this bimodal distribution of samples with CD4+IFNγ+ and separated the population into two groups and investigated its correlation to tumor weights, as shown in **Figure 5A**. Group with CD4+IFNγ^high^ showed smaller tumor weights compared to the group with CD4+IFNγ^low^ cells. This observed tumor weight difference translates into functional relevance based on CD4 IFNγ levels, and hence, we categorized the CD4 IFNγ high expression group as responders and the CD4 IFNγ low expression group as non-responders to the ITI-1001 treatment. In **Figure 5B**, we did unbiased correlation between tumor weight and CD4 IFNγ expression and found a strong negative correlation between the two populations, as seen in **Figure 5A**, mice with CD4 IFNγ high positivity showing smaller tumor burden and mice with CD4 IFNγ low positivity showing higher tumor burden, confirming our conclusion that these groups can be designated as responders and non-responders. **Table 1** shows the numerical data and statistical analysis between responders and non-responders. Furthermore, we characterized these two groups based on other T-cell populations. We found that the responder group showed significantly increased total for CD3+, and CD4+ T cells, while the number of CD8+ T cells was higher but not significantly. T effector memory cells (CD44+CD62L-) were also higher in responders compared with those in non-responders shown in **Figure 5C**. IFNγ and granzyme B+ CD8 T cells were higher in number in the responder population, whereas the CD8+ TNFα+ population was similar in both groups. However, TNFα+ CD4+ T cells were higher in responders compared with those in non-responders, as shown in **Figure 5D**.

## Discussion

GBM, a grade IV astrocytoma, is considered a cold tumor due to the low number of immune cell infiltration within the TME and has low tumor mutational burden, resulting in fewer targetable neoantigens (25). These factors make GBM refractive to most existing checkpoint inhibitor immunotherapies and present a formidable challenge for developing novel treatments (26). The recent failure of checkpoint inhibitor monotherapies was in part attributable to the scarce presence of tumor-specific T cells in the TME. In order to tackle this obstacle, we aimed to boost the immune system with a GBM tumor-targeted approach. We developed ITI-1001, a therapeutic DNA vaccine that contains three immunogenic HCMV antigens (pp65, IE-1, and gB). GBM-specific expression of these HCMV antigens provided a targeted approach, and utilization of our LAMP1-based UNITE platform ensured a strong CD4 and CD8 T cell-driven adaptive response with long-term immunologic memory. As HCMV infects most of the human population, we believe that in an immunologically cold GBM tumor, reeducating the immune system *via* HCMV antigens would result in an effective antitumor immune response leading to increased infiltration and further activation of immune cells in the TME. The use of the three antigens in ITI-1001 minimizes the possibility of antigen escape. Another advantage of using multiple antigens would be to maintain the vaccine’s efficacy even with the known heterogeneous expression of HCMV antigens in patient tumors (7). An alternative to DNA vaccine could be to use immunogenic CD4 and CD8 peptides to induce an immune response, but LAMP1 platform provides such peptides in a physiological manner. And based on our internal work with oncology targets (data not shown), we found that whole or truncated protein fused to LAMP1 generates a much better immune response than that of peptides alone.

ITI-1001 contains HCMV antigens fused to LAMP1 in two plasmid vectors. After injection and subsequent internalization of ITI-1001 pDNA, the glycosylated LAMP1-antigen fusion proteins are expressed in APCs. These fusion proteins are trafficked through the lysosome membrane by LAMP1 and are linked to the internal lysosomal membrane (14). In addition to the MHC-II–lysosome pathway that activates antigen-specific CD4 T cells *via* a Th1 response, LAMP1 also drives the antigens through the MHC-I pathway, leading to cytotoxic CD8 T-cell activation (14, 27, 28). Of note, the plasmid backbone containing CpG islands and the double-stranded plasmid DNA itself further augments the immune response *via* foreign DNA-sensing pathways in the APCs (29).

Results from both *in vitro* and *in vivo* immunogenicity studies of ITI-1001 showed that gB gave the strongest cellular and humoral responses followed by pp65 and then IE-1. Flow cytometry data showed strong CD4 and CD8 T-cell activation, indicating that LAMP1 elicits an immune response *via* both the MHC-II and MHC-I pathways.

To assess vaccine efficacy, we developed an orthotopic syngeneic GBM mouse model expressing HCMV antigens. The CT2A mouse GBM cell line was utilized due to its low tumor burden and refractive nature to immune checkpoint inhibitor monotherapies similar to that of human GBM (25). Interestingly, the ITI-1001 vaccine effectively eliminated GBM in more than 50% of treated mice, suggesting that ITI-1001 generated a strong antigen-specific immune response that could eliminate GBM in these mice and maintain their tumor-free survival for 106 days (study duration) post intracranial injection. This long-term efficacy is even more meaningful considering both the immunologically cold nature of GBM and the aggressive nature of the mouse CT2A GBM cell line.

Tumor cells have a complex relationship with several stromal, vascular, and immune cells that form the TME. The TME plays a crucial role in tumor initiation and growth and in antitumor activities (30, 31). To investigate how ITI-1001 reshapes the TME and to gain insights into the mechanism of action of the vaccine, we evaluated the TME after vaccination. Total T cells (CD3+) showed higher infiltration in the treated tumors compared to control, whose levels were indistinguishable in the two groups. Though CD8 T-cell infiltration did not increase in the treatment group, we observed a significant increase in CD8 T-cell activation, determined by increases in the expression of IFNγ, TNFα, and granzyme B in treated tumors. This strong activation of cytotoxic killer CD8 T cells explains the robust antitumor activity and the corresponding efficacy.

Several recent studies in immunotherapy have shown that in order to have a strong and sustained antitumor immune response, activation of CD8 T cells alone is not sufficient, and additional activation of CD4 T cells is required (32, 33). As a result of our LAMP1 fusion platform, we observed a significant infiltration of CD4 T cells and a concurrent increase in activated CD4 T cells expressing the Th1 pro-inflammatory cytokines IFNγ and TNFα in the treatment group. The preferential activation of CD4 T cells using antigen-LAMP1 fusion proteins has been shown previously in other cancers (10–12, 34). Based on our recent observations using breast cancer model and CD4 depletion studies, CD4 T-cell activation (which results from LAMP1-antigen fusion) was found to be essential for the initial stages of CD8 T-cell activation (14). We hypothesized that ITI-1001 vaccination in our mouse GBM model would result in the generation of activated CD4 Th1 cells that in turn would result in the enhanced activation of CD8 T cytotoxic killer cells by DC licensing and cytokine release (27, 33). CD4 T cells are also known to show direct tumor cytotoxicity and are indirectly involved in the generation of a humoral response (32, 33).

In addition to T-cell infiltration and activation, we also observed significant infiltration of NKT cells in tumors after ITI-1001 vaccination. NKT cells are activated by glycosylated lipids, and the reason behind their increased infiltration in the vaccinated TME is unclear in the absence of any glycosylated lipids in the ITI-1001 vaccine. It remains to be determined whether the heavily glycosylated LAMP1-antigen fusion proteins could be responsible for this infiltration of NKT cells. Interestingly, Duane Mitchell’s group, using single-cell RNA sequencing on human GBM tumors treated with pp65-LAMP1-transfected autologous DCs (35), also found a similar increase in NKT cells in addition to T-cell activation. NKT cells are known to show direct antitumor activity and also help activate CD8 killer T cell *via* DC licensing along with CD4 T helper cells (36, 37). It is noteworthy that although we are unclear on the reason behind the increase in NKT cell infiltration, the similarity in the data with human GBM patients treated with pp65-LAMP1-transfected DCs corroborates the relevance of our preclinical TME data and further supports the therapeutic efficacy of our vaccine in a mouse model.

Upon TME evaluation of immunosuppressive response post vaccination, we observed Tregs to be significantly higher in treated compared to those in the untreated group. Other probable candidates including PD-1, MDSCs, and M1/M2 macrophage populations remained unaltered.

For further confirmation of our TME flow cytometry data showing CD4 and CD8 T-cell activation, we found an increase in Th1 pro-inflammatory cytokine (IFNγ, TNFα, IL-2, and KC/GRO) levels in the tumor lysate. Interestingly, the anti-inflammatory cytokine IL-10 and the Th2 cytokine IL-4 were also upregulated in treated tumors. Elevated IL-10 levels could be explained by the increased number of Treg cells found in the TME in treated tumors and might represent some of the immunosuppressive mechanisms utilized by tumor cells to combat vaccination. It is a known observation that immunosuppressive mechanisms such as expression of cytokine IL-10 is induced to counter the inflammatory reaction induced by the vaccine. In order to increase the efficacy of the vaccine, some researchers have utilized mechanisms that block IL-10 by siRNA, antibody, or by other means (38).

A closer evaluation of the CD4+IFNγ+ population demonstrated two distinct populations based on the number of IFNγ−expressing cells. We separated the treated tumors into two groups based on their CD4 IFNγ expression levels and compared their tumor weights. We found a significant difference between the two groups where the group showing a higher CD4 T-cell IFNγ expression had a lower tumor burden (responders) and the group with a lower CD4 T-cell IFNγ expression had a higher tumor burden (non-responders). To further corroborate the relationship between CD4 T-cell activation and tumor burden, we performed an unbiased correlation analysis of all the treated tumor samples for tumor burden and CD4 T IFNγ+ cell frequency and found a strong negative correlation between these two parameters. This confirmed the significance of CD4 T-cell activation in our vaccine efficacy and also reinforced the responder/non-responder segregation. These two groups were defined further by evaluating other T-cell populations and where we found that CD4 T-cell infiltration and activation including CD4 T-cell expression of TNFα were significantly higher in our responder group. Since CD4 T-cell activation is a critical step in the cellular immune response for LAMP1-fused antigens, this difference is in line with our proposed mechanism of action for the ITI-1001 vaccine. Furthermore, an increased number of total infiltrating CD3+ T cells were observed in the responder group. Also, in correlation with the smaller tumors observed in the responders, we found higher numbers of activated CD8 T cells (IFNγ+ and granzyme B+) in this population, in part explaining the strong antitumor activity of the vaccine. Our objective of identifying and characterizing the responder and non-responder populations was to probe into the lack of CD4 T-cell activation in the non-responders to potentially find ways to further improve the overall efficacy of ITI-1001. In this regard, Tregs were the only cells that were significantly different between the responders and non-responders, but unexpectedly, responders showed higher numbers of Tregs as compared with those in non-responders (**Supplementary Figure S4**). Recent studies have shown antitumor properties of Tregs through their conversion into effector T cells (32, 33). Further studies are needed in our model treated with ITI-1001 to verify such Treg to effector T cell (Teff) conversion. Based on the data from this study, it appears that the non-responding property of tumors to ITI-1001 is independent of M1/M2 macrophages, MDSCs, PD-1 expression and needs further scrutiny.

In summary, based on our preclinical proof-of-concept investigation, ITI-1001 is a potential candidate for human GBM treatment given the robust cellular and humoral response in naive mice, as well as 56% survival efficacy in a GBM mouse model. TME analysis showed strong dependence of CD4 activation to reduce tumor burden and that the vaccine responders showed a high frequency of CD8 T-cell activation. Based on these properties, an Investigational New Drug Application is being submitted with a plan to perform a Phase I clinical trial evaluating the efficacy of ITI-1001 vaccine in the treatment of newly diagnosed GBM patients. Based on the preclinical study outcomes, ITI-1001 can be a new approach for treating GBM patients to reduce tumor burden and prolong progression-free survival.

## Data Availability Statement

All data relevant to the study are included in the article or uploaded as online **Supplementary Information**. Any clarification on the data, protocols, or statistical methods used will be available on request.

## Ethics Statement

All animal studies were reviewed and approved by the Institutional Animal Care and Use Committee.

## Author Contributions

AA designed, performed, analyzed the studies and wrote the article. JM, YJ, and MC performed the studies. TC and TH supervised the studies and reviewed the article. All authors contributed to the article and approved the submitted version.

## Funding

This study received funding from Immunomic Therapeutics. The funder was not involved in the study design, collection, analysis, interpretation of data, the writing of this article or the decision to submit it for publication.

## Conflict of Interest

AA, JM, YJ, MC, TC, and TH are employees of Immunomic Therapeutics, and TH is also a shareholder.

## Publisher’s Note

All claims expressed in this article are solely those of the authors and do not necessarily represent those of their affiliated organizations, or those of the publisher, the editors and the reviewers. Any product that may be evaluated in this article, or claim that may be made by its manufacturer, is not guaranteed or endorsed by the publisher.

## Supplementary Material

Supplementary Figure 1 Western for ITI-1001 antigens, pp65, IE-1 and gB using LAMP-specific antibody. Lane 1- Molecular weight marker, Lane 2, 293T untransfected, non-deglycosylated cell lysate, Lane 3, 293T untransfected, deglycosylated cell lysate. Lane 4, Human LAMP1non-deglycosylated protein, Lane 5, Human LAMP1deglycosylated protein, Lane 6, ITI-1001 transfected 293T, non-deglycosylated cell lysate, Lane 7 ITI-1001 transfected 293T, deglycosylated cell lysate.

Supplementary Figure 2 ELISpot data for IL-4. IL-4 ELISpot data with antigen-specific peptides for pp65, IE-1, gB. Data was not analyzed further due to the low number of spots.

Supplementary Figure 3 Investigating other immune cells in the TME upon ITI-1001 vaccination. **(A)** Flow cytometric data for mo MDSCs and PMN MDSCs in the TME with and without ITI-1001 vaccination. **(B)** Flow cytometry data for PD1 expression of CD8 T cells. C. Flow cytometry data for M1 (Arginase1+) and M2 (INOS+) macrophages. All the populations are denoted as cell numbers per gram of tumor. p values from Student’s t-test are denoted to signify the statistical differences between the control and treated tumors.

Supplementary Figure 4 Frequencies of regulatory T cells in responders and non-responders. Graph plotted for number of T-regs per gm of tumor in responders and non-responders. Segregation of responders and non-responders is based on the levels of CD4 IFNg. p values from Student’s t-test are denoted to signify the statistical differences between the responders and non-responders.

Supplementary Figure 5 Gating strategy for TME immune cells and FMO controls. **(A)** representative image of gating strategy for T activation, PD-1 expression and T-regs, NK cells and macrophages. **(B)** FMO controls for the various antibodies.
