# Supplementary material for: Development and Characterization of an HCMV Multi-Antigen Therapeutic Vaccine for Glioblastoma Using the UNITE Platform
Source: Front Oncol. 2022 May 16;12:850546. doi: 10.3389/fonc.2022.850546 (PMC9149224; doi:10.3389/fonc.2022.850546)
Supplement: Supplementary Figure 1 — Western for ITI-1001 antigens, pp65, IE-1 and gB using LAMP-specific antibody. Lane 1- Molecular weight marker, Lane 2, 293T untransfected, non-deglycosylated cell lysate, Lane 3, 293T untransfected, deglycosylated cell lysate. Lane 4, Human LAMP1non-deglycosylated protein, Lane 5, Human LAMP1deglycosylated protein, Lane 6, ITI-1001 transfected 293T, non-deglycosylated cell lysate, Lane 7 ITI-1001 transfected 293T, deglycosylated cell lysate. [file Presentation_1.pptx]

## Slide 1
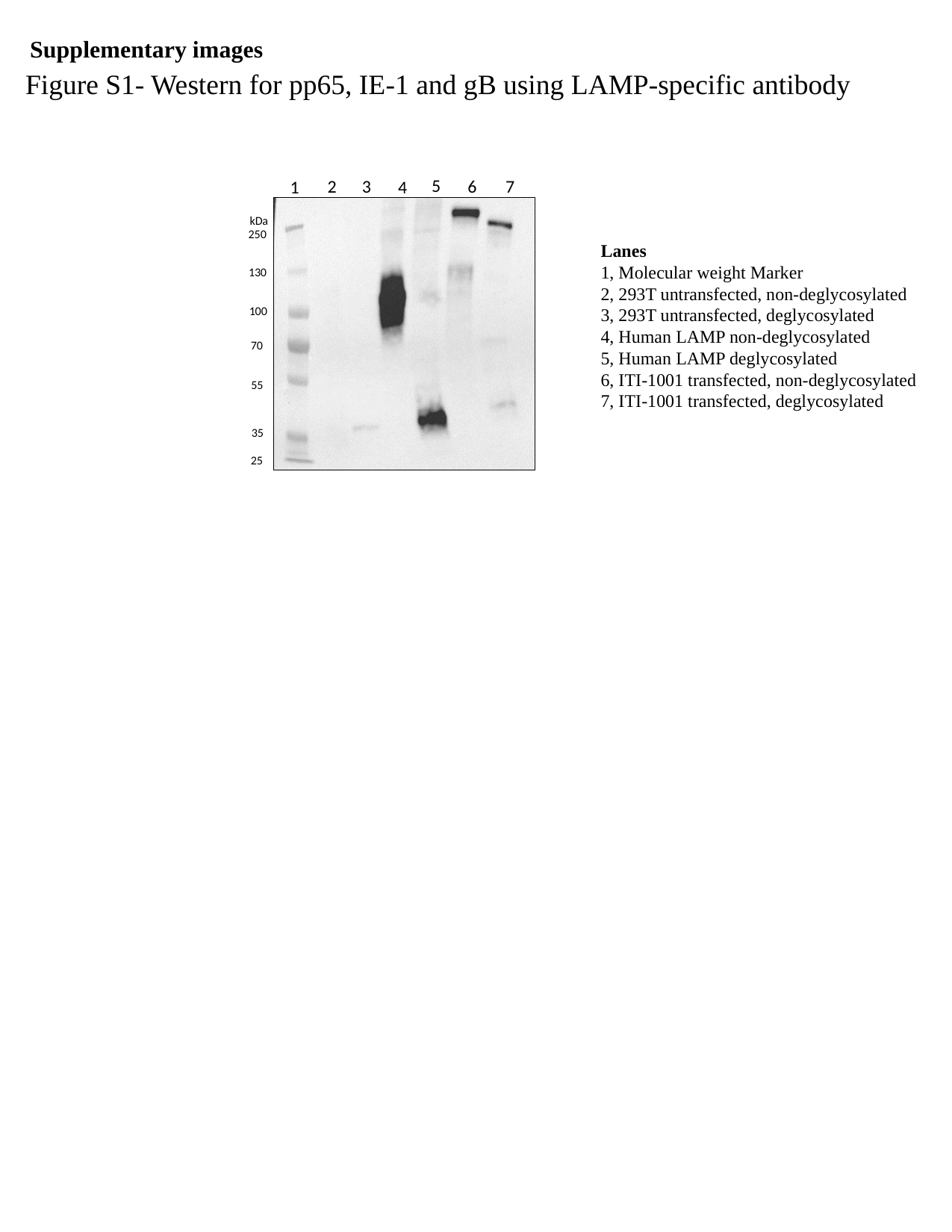

Supplementary images
Figure S1- Western for pp65, IE-1 and gB using LAMP-specific antibody
5
6
2
7
3
4
1
kDa
250
Lanes
1, Molecular weight Marker
2, 293T untransfected, non-deglycosylated
3, 293T untransfected, deglycosylated
4, Human LAMP non-deglycosylated
5, Human LAMP deglycosylated
6, ITI-1001 transfected, non-deglycosylated
7, ITI-1001 transfected, deglycosylated
130
100
70
55
35
25

## Slide 2
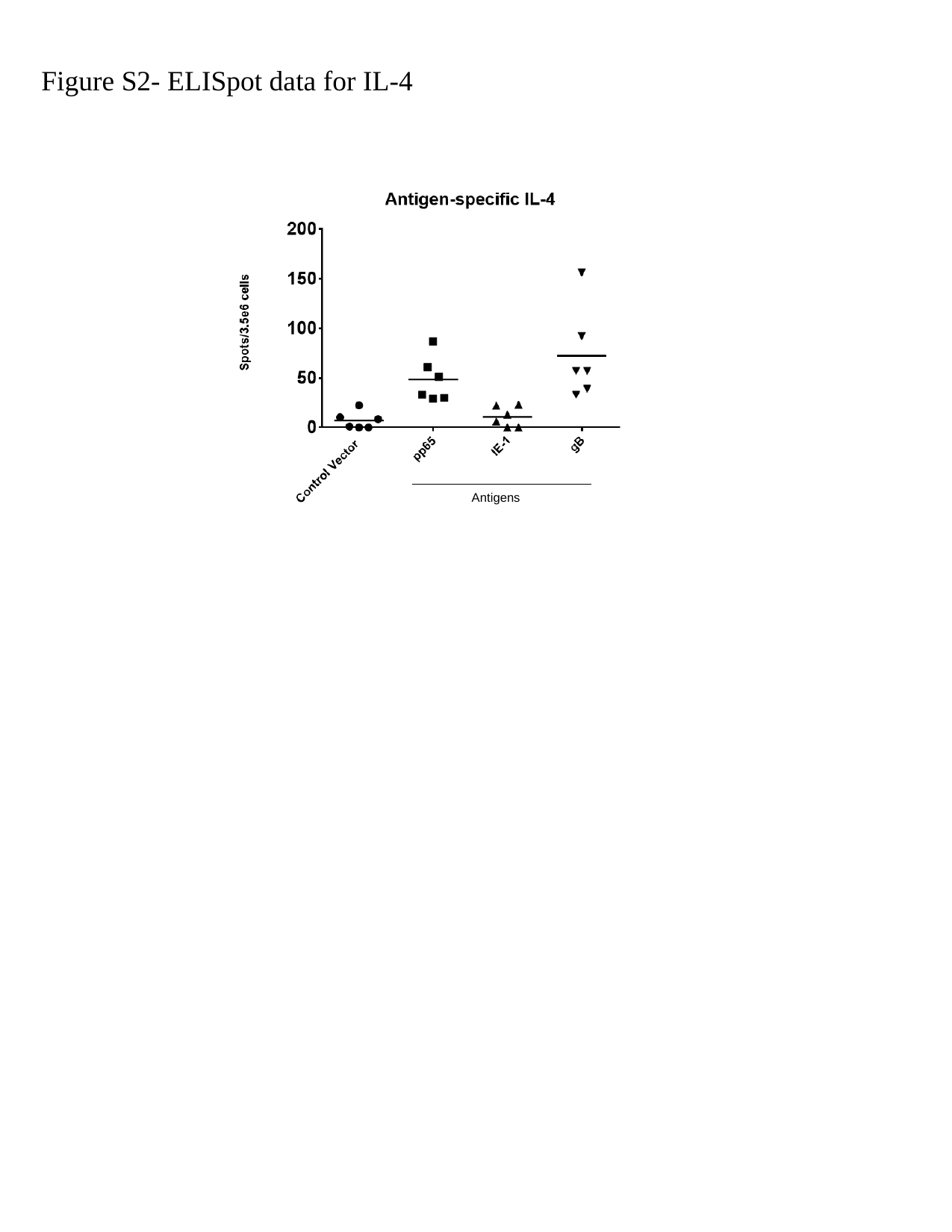

Figure S2- ELISpot data for IL-4
Antigens

## Slide 3
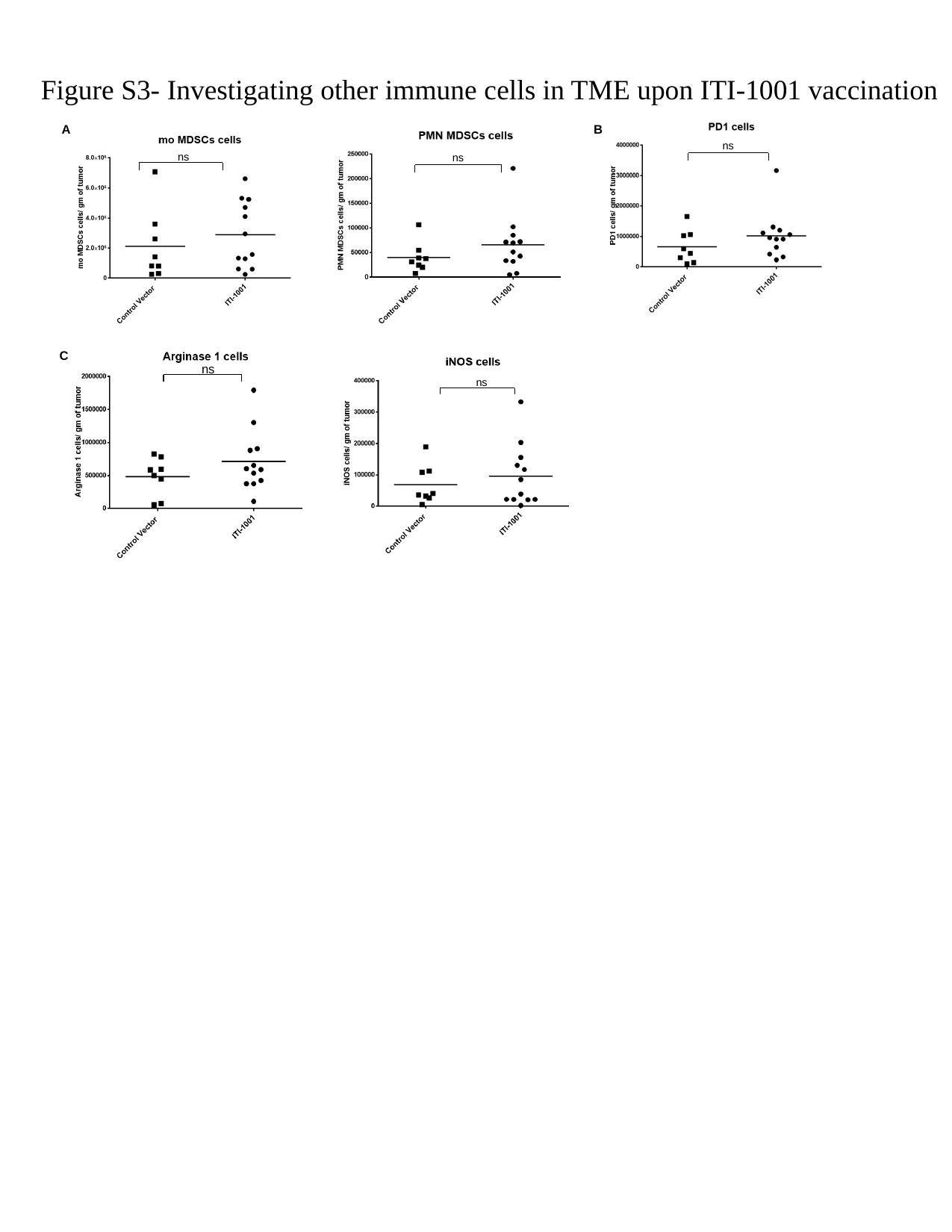

Figure S3- Investigating other immune cells in TME upon ITI-1001 vaccination
A
B
ns
ns
ns
C
ns
ns

## Slide 4
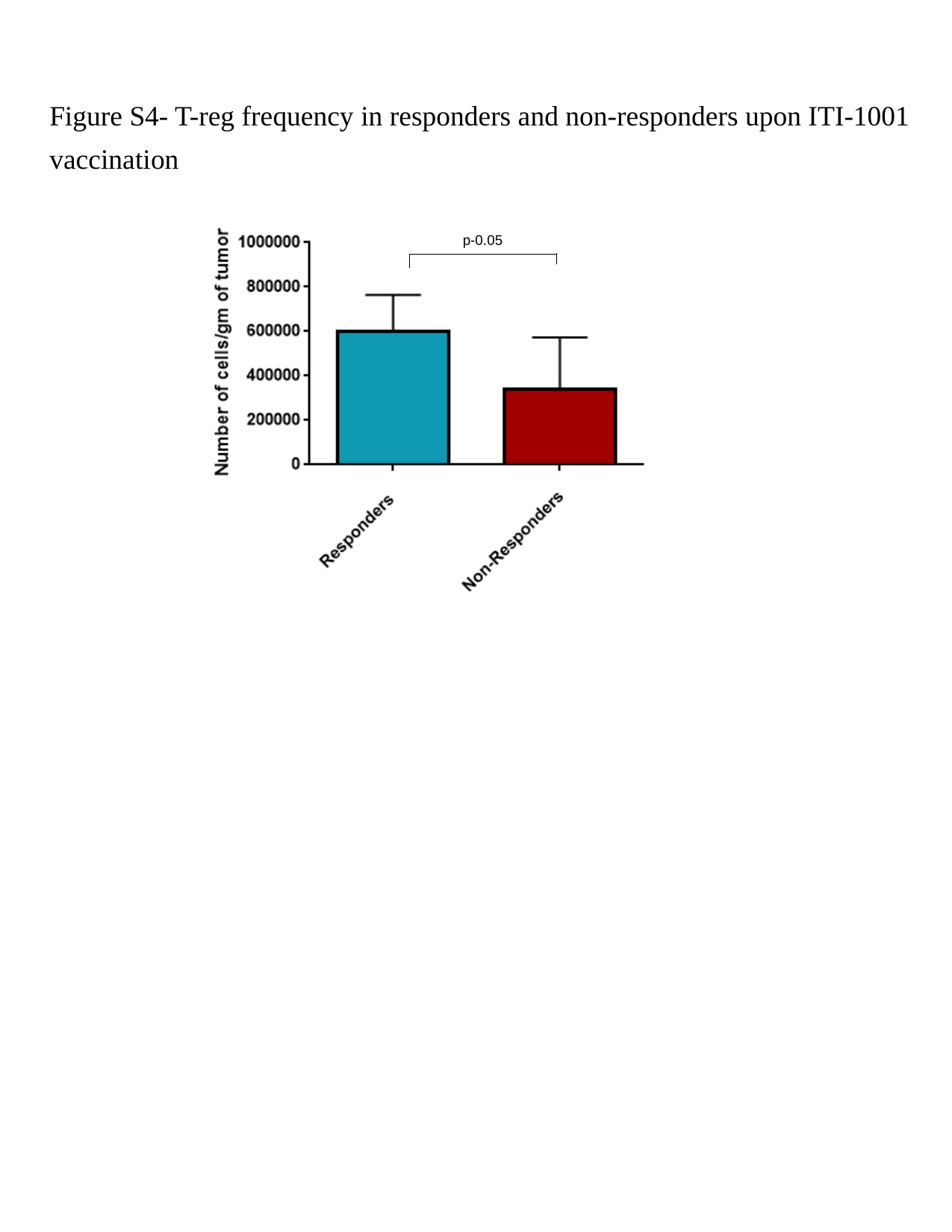

Figure S4- T-reg frequency in responders and non-responders upon ITI-1001 vaccination
p-0.05

## Slide 5
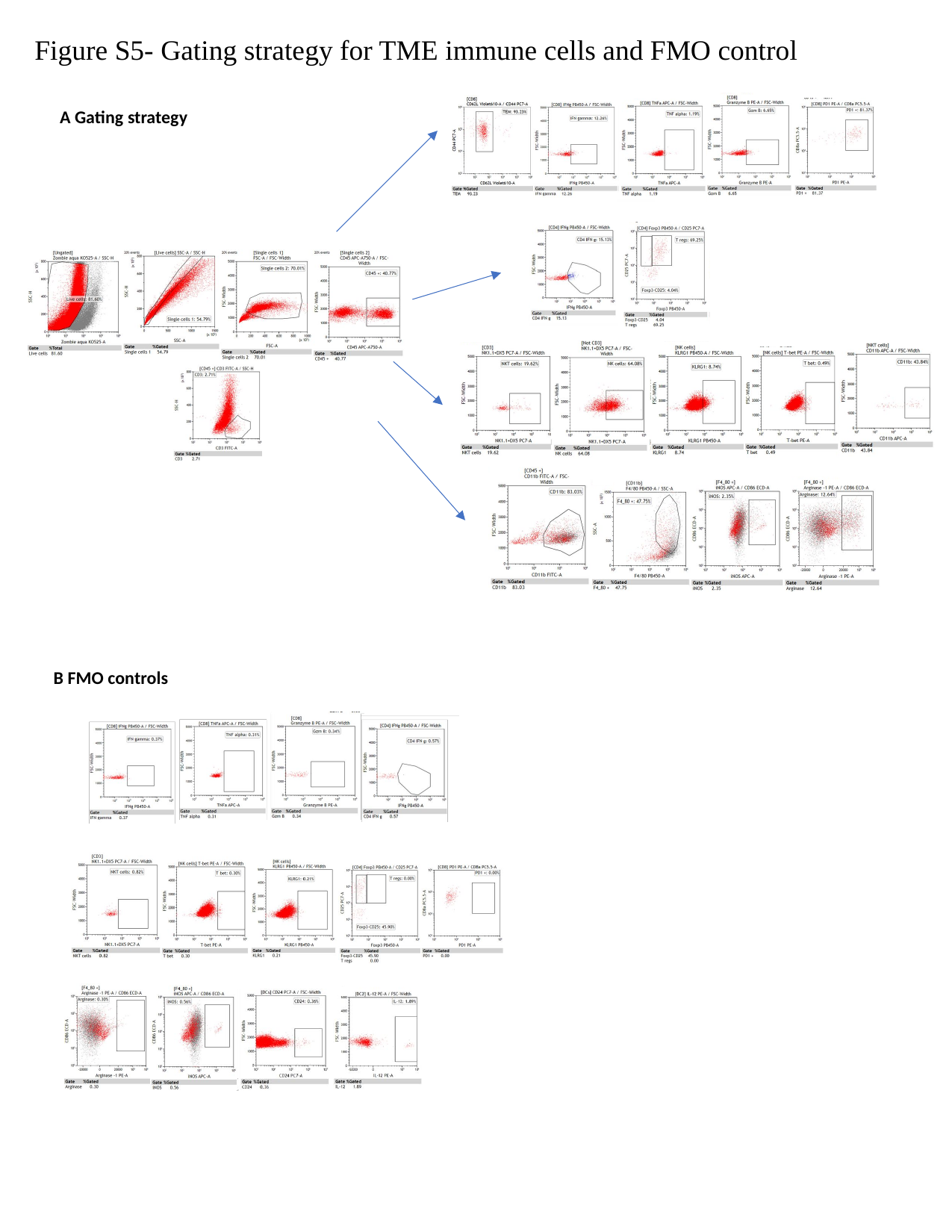

Figure S5- Gating strategy for TME immune cells and FMO control
A Gating strategy
B FMO controls

## Slide 6
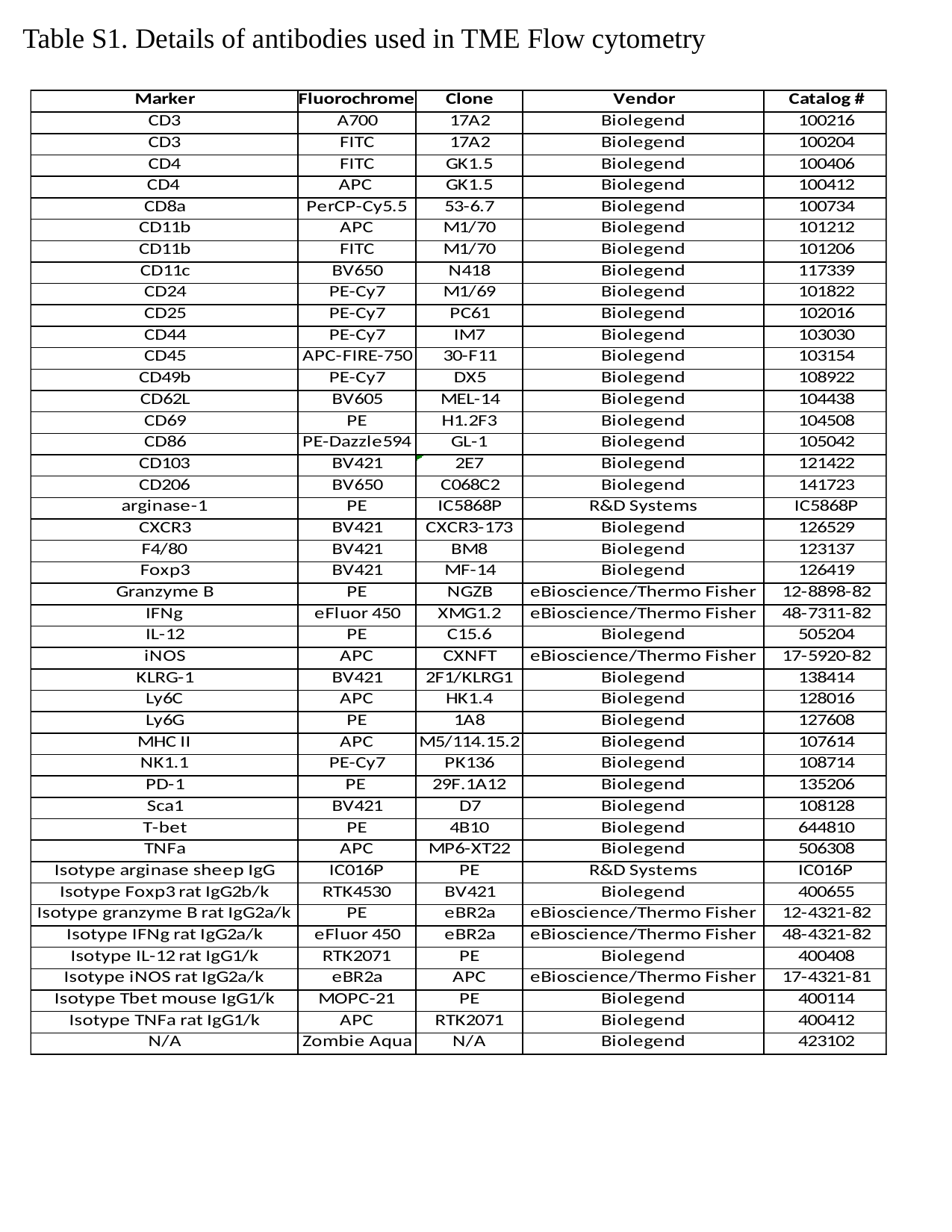

Table S1. Details of antibodies used in TME Flow cytometry
